# Survival and haematological recovery of children with severe malaria transfused in accordance to WHO guidelines in Kilifi, Kenya

**DOI:** 10.1186/1475-2875-7-256

**Published:** 2008-12-16

**Authors:** Samuel O Akech, Oliver Hassall, Allan Pamba, Richard Idro, Thomas N Williams, Charles RJC Newton, Kathryn Maitland

**Affiliations:** 1 Centre for Geographic Medicine Research (Coast), Kenya Medical Research Institute, PO Box 230, Kilifi, Kenya; 2 Liverpool School of Tropical Medicine, Liverpool, L3 5QA, UK; 3 Department of Paediatrics and Child Health, Mulago Hospital/Makerere University Medical School, Kampala, Uganda; 4 Nuffield Department of Medicine, John Radcliffe Hospital, University of Oxford, Oxford, UK; 5 Neurosciences Unit, Institute of Child Health, The Wolfson Centre, Mecklenburgh Square, London, WC1N 2AP, UK; 6 Clinical Research Unit, London School of Hygiene and Tropical Medicine, London, UK; 7 Department of Paediatrics and Wellcome Trust Centre for Clinical Tropical Medicine, Faculty of Medicine, Imperial College, Norfolk Plac3e, London, W2 1PG, UK

## Abstract

**Background:**

Severe anaemia requiring emergency blood transfusion is a common complication of malaria in children. To ensure access for urgent blood transfusion, the World Health Organization has developed clear guidelines with haemoglobin thresholds prevent unwarranted transfusion,. Few studies have reported outcome and haematological recovery of children with severe malaria where transfusion practice complies with WHO recommendations.

**Methods:**

A prospective observational study of survivors of severe and complicated malaria transfused in accordance with WHO guidelines. Children were invited for review at one month post-discharge. Non-attendees were traced in the community to ascertain survival.

**Results:**

Outcome was assessed in 213 survivors. Those transfused were younger, had a higher base deficit, mean lactate levels and a higher prevalence of respiratory distress. As expected mean admission haemoglobin (Hb) was significantly lower amongst transfused [5.0 g/dL SD: 1.9] compared to non-transfused children [8.3 g/dL SD: 1.7] (p < 0.001). At discharge mean Hb was similar 6.4 g/dL [SD: 1.5] and 6.8 g/dL [SD: 1.6] respectively (p = 0.08), most children remained moderately to severely anaemic. At one month follow up 166 children (78%) returned, in whom we found no differences in mean Hb between the transfused (10.2 g/dL [SD: 1.7]) and non-transfused (10.0 g/dL [SD: 1.3]) survivors (p = 0.25). The major factors affecting haematological recovery were young age (<24 months) and concomitant malaria parasitaemia; Hb being 8.8 g/dL [SD: 1.5] in parasitaemic individuals compared with 10.5 g/dL [SD: 1.3] in those without (p < 0.001).

**Conclusion:**

This data supports the policy of rational use of blood transfusion, as proposed in the WHO guidelines, for children with anaemia in areas where access to emergency transfusion is not guaranteed. We have provided empirical data indicating that transfusion does not influence superior recovery in haemoglobin concentrations and therefore cannot be justified on this basis alone. This may help resolve the disparity between international policy and current clinical practice. Effective anti-malarial treatment at discharge may prevent reoccurrence of anaemia.

## Background

Annually approximately two billion people are exposed to *Plasmodium falciparum *resulting in over 500 million clinical cases and about one million deaths predominantly in children less than five years living in the sub-Saharan Africa (SSA) [1]. Malaria complicated with severe anaemia (Hb<5 g/dL) is an important public health problem in this patient population resulting in major life threatening complications and is a major cause of mortality [2-4]. Anaemia secondary to malaria accounts for up to 70% of all prescribed transfusions in malaria endemic SSA [5-7]. Previous studies have revealed that severe anaemia secondary to malaria without any other complications leads to about 1% mortality, however, this rises to 16% when complicated with respiratory distress (severe, symptomatic anaemia) and over 30% when both respiratory distress and coma also present[6].

For children with severe, symptomatic anaemia, it is now recognized that an urgent blood transfusion is life saving[2]. However, in the many African hospitals blood banking facilities are inadequate [8] and urgent transfusions are often not possible [9]. In these circumstances children have to wait while a replacement donor is found and over 60% of deaths in children with severe malaria anaemia occur before a transfusion can be given[2,10]. The majority of transfusions are, therefore, received by children with stable, uncomplicated anaemia, for whom the acute benefits of transfusion are unproven [9,11], One study in Tanzania reported up to 50% avoidable transfusions[11]. Often, what is is not considered is that the risks of transfusion may outweigh the benefits, since blood transfusions continue to be a major source of preventable HIV infection [12]and other transfusion-transmissible infections [2,3,13,14]. These risks are over and above those due to transfusion reactions[14] and often not detected due to poor haemovigilence. It is, therefore, important to carefully delineate a group of patients who need a blood transfusion.

The World Health Organization (WHO) guidelines, which are largely based on expert opinion [15-17] and a few previous studies [18], encourage rational use of blood. These guidelines recommend that transfusion be reserved only for children with absolute haemoglobin (Hb) of ≤ 4 g/dL (profound anaemia) or haemoglobin of 4–5 g/dL plus respiratory distress in malaria endemic areas and a higher cut off of 7 g/dl in areas of low malaria transmission[19]. Adherence to the targeted use of transfusion is hampered by the lack of clinical evidence that this policy is safe in both the short and long term[20]. As a result many children, who do not fulfil criteria for transfusion, continue to receive transfusions in the belief that both short and longer term outcome and haematological recovery is improved.

Whilst there is compelling evidence to support use of blood in children with profound anaemia and severe anaemia with respiratory distress[2,3] evidence supporting transfusion avoidance in the group with severe anaemia (Hb 4–5 g/dL) without respiratory distress or moderately severe anaemia (Hb < 7 g/dl) with life-threatening complications [2,21,22] is less convincing. Further, there are conflicting findings on the effect of transfusion on haemoglobin recovery after discharge[2,21,22], which may help inform a decision on the choice of whether or not to conservatively manage these children. Setting aside considerations of availability and safety, often overlooked are the economic considerations of blood transfusion, with an average cost of 30 US dollars in most localities, rising up to 50 US dollars where voluntary non-remunerated donors are used; most of these costs are recovered from the patients or their families[23].

We have previously reported in-hospital outcome in children with severe malaria including those with and without symptomatic anaemia [24-27]. In this current study we report haematological recovery at discharge, one month post-admission and longer term survival in children admitted with severe malarial anaemia complicated by respiratory distress transfused in accordance to WHO guidelines.

## Methods

### Setting

The study was conducted between May 2002 and January 2005 at the Kenya Medical Research Institute, Kilifi District Hospital, Kenya. Patients attend this hospital from a well mapped Demographic Surveillance Site (DSS) in which a census is conducted three times a year. Malaria is endemic in Kilifi District, where falciparum parasitaemia at the time of the study was present in over 30% of children in the community and 46% of hospital admissions[4,28]. Although many children are admitted with a primary diagnosis of severe malaria complicated by some degree of anaemia, the etiology of anaemia in this population is complex[4,29]. The study area is one of the poorest in Kenya with 40% of children <5 years having anthropometric measures of undernutrition, half with biochemical markers of iron deficiency, 14% of children within the DSS have sickle cell and almost 60% have α^+ ^thalassemia deletion genotypes[13].

### Participants

Demographic and clinical data were recorded on admission using a standard proforma. Children were eligible for inclusion in this study if they manifested all of the following features: *P. falciparum *parasitaemia (at any density), a clinical feature of severe malaria (prostration, coma or respiratory distress) and metabolic acidosis (base deficit of >8). Such children are at the greatest risk of death, including the subgroup of children with symptomatic severe malaria anaemia (Hb<5 g/dl in association with signs of cardiorespiratory compromise).

### Standard treatment

In accordance with WHO guidelines whole blood transfusion (20 mls/kg of whole blood) was administered (without diuretics) for children admitted with or subsequently developing a Hb <4 g/dL or 4–5 g/dL if associated with respiratory distress. Those with these features at admission were transfused immediately (early transfusion group) while those without these features at admission were transfused later if they developed these features (delayed transfusion group). Children with features of decompensated shock meeting above transfusion criteria (early transfusion group) were administered pre-transfusion management with 10 mls/kg fluid while awaiting transfusion[26]. Children with a Hb>5 g/dl and respiratory distress were enrolled into intervention trials and received volume expansion with saline, albumin or gelofusine [24-27]. Following resolution of features of severe malaria and once children were able to take and retain oral medication, anti-malarial treatment was completed with a full course of oral anti-malarial therapy. In accordance with local national guidelines, the children received a single dose of sulphadoxine-pyrimethamine (SP) up to December 2003, and SP plus amodiaquine thereafter when the national anti-malarial policy had changed.

### Discharge and follow-up

Discharge from hospital was consequent upon establishing both clinical and parasitological recovery (malaria slide negative). Children were discharged with any level of haemoglobin ≥ 4 g/dL as long as they did have respiratory distress. At discharge all survivors received a course of iron sulphate (20 mg/kg/day in three divided doses for 28 days which was continued to complete three months), folate (2.5/5 mg once day for body weight <10/>10 kg respectively for 28 days and continued to complete three months), and mebendazole (100 mg twice daily for three days). Children reviewed before 21 days or after 35 days were excluded from the primary analysis of haematological recovery due to temporal effects on haemoglobin recovery, but included in survival analysis. Follow-up review included a full clinical assessment, anthropometric measurement, full blood count, and a malaria slide. Investigation of parasite genotyping was not performed (to distinguish re-infection from recrudescence). Survival of study children was also obtained by linking the clinical data to the census data routinely done in the DSS reference to find out the long term progress of the study participants. In this study, information from census done in May 2005 was used.

### Data analysis

Data was analysed using Stata version 8.2. Patients were retrospectively classified as transfused or not transfused and by compliance with follow-up. In children attending follow-up within one month of post discharge admission, discharge and follow-up characteristics of children who were transfused according to WHO guidelines were compared to those of their relatively less sick counterparts who did not received blood transfusion, because they did not fulfil the WHO criteria, by comparing means and standard deviations for continuous variables and Chi^2 ^or Fisher's exact test for proportions. The haemoglobin at follow-up was further categorized into <9.3 g/dL (WHO criteria for anaemia) and >9.3 g/dL with those who failed to attain a Hb of 9.3 g/dL classified as anaemic according to WHO criteria[19]. Mantel-Haenszel methods were then used to calculated crude odds ratios for association of various factors with anaemia at follow-up (i.e. Hb <9.3 g/dL) and a logistic regression analysis done to determine independent associations. All variables with P-value < 0.25 at univariate analysis were included in a logistic regression analysis to identify independent determinants of haemoglobin recovery and only those with a P value < 0.05 were retained in the model. An *a priori *decision was made to include age in each model due possible confounding effect of age on incidence of anaemia in children with malaria. Formal survival analysis was not performed, instead survival was described as the proportion of cases that were ascertained at discharge, follow-up and long-term follow-up by DSS.

## Results

Of the 241 participants in three intervention studies [24,26,27]) admitted with severe malaria complicated by metabolic acidosis, 213 (88%) survived to discharge and 166 (74%) attended follow-up. In hospital outcome has been previously reported in detail[24,26,27] but is summarized briefly for the whole cohort (n = 241) with respect to early and delayed transfusion. The participants included 65 (27%) children with profound anaemia (Hb ≤ 4 g/dL) or Hb 4–5 g/dL with respiratory distress (symptomatic severe malaria anaemia-SSMA), 14 (6%) with Hb between 4–5 g/dL without respiratory distress, 74 (30%) children with moderate acidosis (base deficit 8–15) and Hb>5 g/dL at admission and 88 (37%) children with severe acidosis (base deficit > 15) and Hb>5 g/dL at admission

### Blood transfusion practice

#### Early transfusion group

Out of 241 children studied 65 (27%) had SSMA requiring urgent transfusion according to WHO guidelines: 64 (98%) received a blood transfusion while one child died before transfusion could be obtained. Mortality was low, only five (7.7%) children in this group died: four of these children were in coma at admission and died within eight hours of admission. All cases had a cardiorespiratory arrest.

#### Delayed transfusion group

In addition 45 (18.6%) children received a whole blood transfusion later during the course of their admission after fulfilling the criteria outlined by WHO. Among the fourteen children initially with severe anaemia (Hb of 4–5 g/dL), but without respiratory distress, ten (71%) were eventually transfused because they developed impaired consciousness and/or respiratory distress. For children with Hb> 5 g/dl with moderate or severe metabolic acidosis (base deficit >8 and >15 respectively) who were initially treated for shock with volume expansion, 5/74 (7%) and 30/88 (34%) children respectively also went on to receive a whole transfusion due to a fall in haemoglobin below 5 g/dL. No differences in the proportion requiring delayed transfusion were found between the different resuscitation fluids. Four children (8.8%) in the delayed transfusion group died; all had been admitted in deep coma. No cases developed pulmonary oedema or a transfusion reaction.

### Haematological recovery of survivors

Of the 213 survivors, 166 (78%) patients returned for follow-up assessment: 158 (74%) children were reviewed between 21–35 days post discharge and eight (4%) were reviewed outside this interval. Out of these eight children, three attended earlier due to malarial fever (2) or malnutrition (1) and five were well, but attended on the wrong date. Haematological recovery was assessed in 158 survivors who subsequently attended follow-up within 22 to 35 days post-admission

### Baseline characteristics

Baseline data are summarized in Table 1 with respect to transfusion status. The mean admission Hb was 5.0 g/dL [SD 1.9] in those receiving a blood transfusion while in those not transfused, the mean Hb was 8.3 g/dL [SD 1.9]. Those transfused were younger, median age 25 months (SD 14–35) versus 36 months (SD 27–47); P < 0.001) and had poorer nutritional status, mean mid-upper arm circumference (MUAC) 13.6 cm [SD: 1.3] versus 14.5 cm [SD: 1.5]; (P < 0.001). Geometric mean parasitaemia was similar in the transfused group 2.6 × 10^5 ^(95% Reference Range (RR) 1.7, 3.5) and non-transfused group 2.3 × 10^5 ^(95% RR 1.7, 2.9) (p = 0.62) as were sex, prevalence of hypoglycaemia or mean corpuscular volume (MCV) (Table 1). However, presence of respiratory distress, base deficit and mean lactate levels were all significantly higher in the group of patients who received a blood transfusion (Table 1).

**Table 1:** Baseline and follow-up characteristics of participants

**Characteristic**	**Survivors (n = 158)**		
	Transfused	Not transfused	P

N (%)	61 (39)	97(61)	

Age in months (median, IQR)	24(14–35)	31(21–43)	0.001

Proportion < 24 months (%)	24	48	0.002

Male sex (n, %)	30(49)	52(57)	0.59

Admission duration (days) (median, IQR)	5(4–8)	4(3–6)	0.13

Review interval (days) (median, IQR)	28 (25–31)	28(27–31)	0.88

Respiratory distress (n, %)	46(76)	54(56)	0.001

Lactate (mmol/L, mean SD)	5.8(4.5)	4.8(3.7)	0.06

Hypoglycaemia (<2.2 mmol/L) (n, %)	10(16)	21(22)	0.239

Severe acidosis (Base deficit >15) (n, %)	37(60)	45(46)	0.03

*Haematological variables*			

Admission Hb (mean, SD)	5.0 (1.9)	8.3(1.7)	0.001

MCV (mean, SD)	75.6(6.3)	74.2(6.8)	0.09

MUAC (mean, SD)	13.8(1.3)	14.4(1.5)	0.02

Discharge Hb (mean, SD)	6.4 (1.5)	6.8(1.6)	0.08

Follow-up Hb (mean, SD)	10.2(1.7)	10.0(1.3)	0.25

### At hospital discharge

Significant changes were observed in mean Hb between admission and discharge which increased on average by 1.4 g/dl (SE 0.2) in those receiving a blood transfusion and fell by a mean of 1.5 g/dl (SE 0.1) of Hb in the non-transfused group. Although there were clear differences in Hb at admission there were no differences in discharge Hb in the two groups (F = 1.4; P = 0.23) (Table 1). At discharge from hospital, 37/213 (17%) remained severely anaemic (Hb 4–5 g/dL) and 100 (47%) children had moderately severe anaemia (Hb 5–7 g/dl). All survivors were aparasitaemic at time of discharge.

### At follow-up review

**Figure 1:**
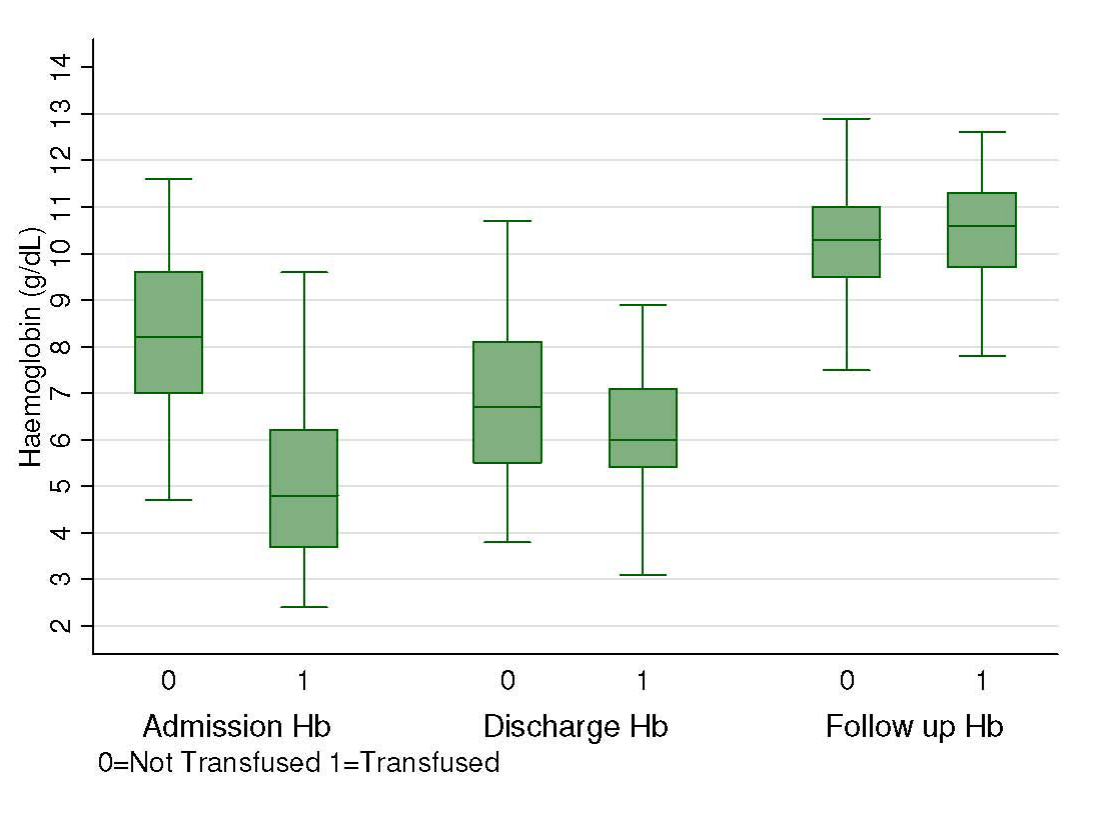
**Post discharge haemoglobin recovery in children with severe malaria according to transfusion status**. Admission mean haemoglobin was lower in those transfused than those not transfused (p = 0.001), but there were no significant differences in the mean haemoglobins at discharge (p = 0.08) and at follow-up (p = 0.25).

Haematological recovery was identical in both groups with no significant differences in the mean follow-up haemoglobin between those transfused and those not transfused (p = 0.25) (Table 1). Overall, there was an 46% mean increase of post-admission Hb at follow up, with 74% attaining an effective haematological recovery (Hb >9.3 g/dL) and 89/158 (56%) achieving values greater than 10 g/dl at follow up (Figure 1). For the 47 (22%) non-attendees, there were no differences in the mean haemoglobins at admission (p = 0.16), discharge (p = 0.34), and transfusion status or other marker of severity from those that attended follow-up.

The presence of malaria parasitaemia at follow-up and young age were the only factors independently associated with follow-up haemoglobin. Nutritional status (determined by mid-upper arm circumference (MUAC) and weight), mean corpuscular volume (MCV) and review interval did not have a significant influence on the follow-up haemoglobin.

### Malaria parasitaemia at follow-up

**Figure 2:**
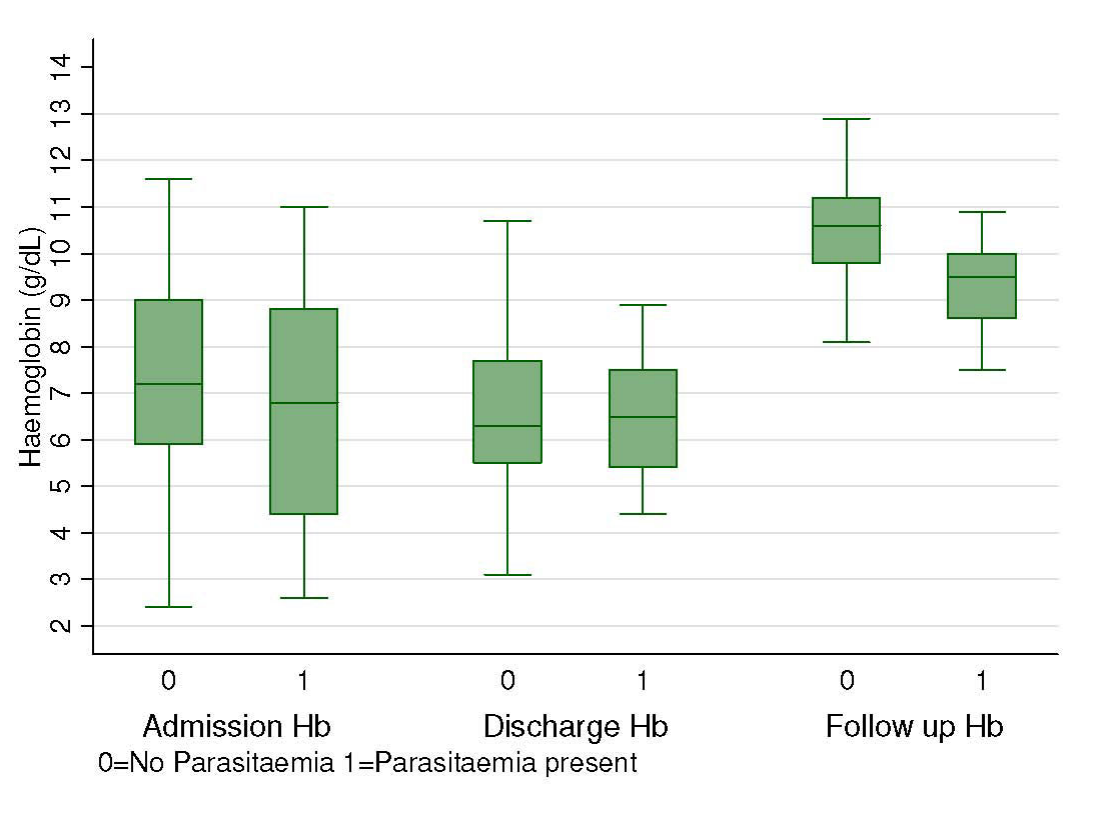
**Post discharge haemoglobin recovery in children with severe malaria according to presence or absence of parasitaemia at follow up**. Children with malaria parasitaemia at follow-up had similar mean haemoglobins to those without parasitaemia at admission and discharge. However, the mean haemoglobins at one month follow-up were higher in those without parasitaemia (10.5 g/dL) than those with parasitaemia (8.8 g/dL) (p = 0.001).

Presence of malaria parasitaemia was found to be the most significant determinant of haemoglobin recovery in patients following admission with severe malaria (Figure 2). For those with parasitaemia, mean haemoglobin was 8.8 g/dL (SD: 1.5) compared to a mean of 10.5 g/dL (SD: 1.3) in aparasitaemic children; P < 0.001. This was not influenced by mean haemoglobin at admission or discharge, age, sex, review interval, MCV and MUAC as they were not significantly different in the two groups. Of the patients with parasitaemia at follow-up, 15/35 (43%) were transfused while 20/35(57%) were not transfused (P = 0.48). The odds ratio of a low haemoglobin (Hb <9.3 g/dL) in cases with parasitaemia compared to no malaria was 4.00 (1.61–8.42) (p = 0.002) and this remained significant even after controlling for age, treatment received, MUAC, MCV and sex (p = 0.001). Anti-malarial treatment used at discharge had a significant influence on the follow-up mean haemoglobin. Of the 158 patents included, 27/158 (17%) had sulfadoxine-pyrimethamine(SP)-amodiaquine combination while 131/158 (83%) had SP alone. Children who had a combination therapy with SP-amodiaquine had significantly higher mean haemoglobin (10.9 g/dL SD 1.4) at the time of follow-up compared to those who had SP alone (9.9 g/dL SD1.5) (p = 0.005).

### Age

Age <24 months was found to be a significant determinant of haemoglobin at admission (p < 0.001), discharge (p = 0.008), and at follow-up (p = 0.01). Younger children (age <24 months) had a lower Hb at admission (F = 15; p < 0.001), discharge (F = 5; p = 0.008) and at review (F = 4; p = 0.03). Children under 24 months old accounted for 31/61 (51%) of all transfusions; 14 given for SSMA and 17 delayed transfusions.

### Follow-up in the community

Medium term survival was ascertained in the 166/213 (78%) patients who returned to follow-up, and the demographic surveillance was used to trace survival in those who failed to attend follow-up review. Of the 47 non-attendees 32 (68%) came from within the study area, of whom we could confirm longer term survival on 30. Twenty one were reported to be still alive and living in the census area, 9/32 were still alive when they migrated out of the study area and two were not traceable. This increases the overall medium term survival to 196 (92%) children with severe and life threatening malaria who were managed conservatively with respect to blood transfusion in accordance with WHO transfusion guidelines.

## Discussion

This study demonstrates a number of important points. First, if provides evidence that the WHO policy for rationale use of blood transfusion in children with severe malaria is justified. In these children both immediate and longer-term child survival were optimized by strictly limiting blood transfusions to a high-risk group with urgent need of transfusion, and preventing unnecessary exposure of those not warranting transfusion to the hazards of blood transfusion.

Secondly, the study demonstrates that despite fall in haemoglobin during admission for those who are less sick and do not fulfill the WHO criteria for transfusion, they have satisfactory haematological recovery even if they are discharged with moderate anaemia. They also do not have increased risk of mortality either during admission or at discharge. The absence of deaths among those children who did turn up for one month follow-up but resident in the study area at least six months after the last patient was recruited allays fears of bias due to loss of follow-up.

Thirdly, this study shows that complete parasitological recovery is very important for a satisfactory hematological recovery. Once malaria parasitaemia is cleared the Hb recovers rapidly with haematinic supplementation in this study. We found that haematological recovery at one month follow-up of these children was remarkable: 74% attaining an effective haematological recovery (Hb >9.3 g/dL) and 89/158 (56%) achieving values greater than 10 g/dl. Neither blood transfusion nor nutritional status affected mean Hb at follow-up. Although not directly addressed by this study we suspect that anti-malarial drug resistance was a major factor threatening hematological recovery and is supported by similar studies in Kenya and Tanzania [3,22]. Together, these emphasize the need for an effective anti-malarial treatment to aid recovery. Although we have limited data suggesting that a better haematological recovery on the combination therapy of SP-amodiaquine than those on SP alone, this finding underscores the value of effective treatment for good haematological recovery rather than blood transfusion. The availability of effective anti-malarial combination therapies will not only result in less malaria and economic savings of inpatient treatment but may help to resolve some of the current inadequacies of blood banking services.

The relative severity of anaemia in those less than 24 months is consistent with findings in other studies and is likely to be a reflection of poor immunity to malaria [3,4,15]. A study done in The Gambia showed a comparatively better recovery in children with Hb of 4–5 g/dL receiving iron treatment than those who received a blood transfusion [30]. While this difference could be due to bone marrow suppression secondary to a blood transfusion, children in Kilifi have a high prevalence of nutritional deficiencies[13,31,32] and the similar haematological recovery seen in our study for the two groups when both are administered haematinics could be due to the correction of iron deficiency.

In severe malaria this study provides some support that blood transfusion can be avoided, and that while the majority of children were moderately to severely anaemic on discharge, most demonstrated a good haematological recovery. This latter finding provides encouraging justification for investigation for the use of the low volume umbilical cord blood as an alternative source of blood for transfusion[33]. Studies on the use of artificial haemoglobins which have never been tested in this population of patients but could provide immediate benefits to oxygen carrying capacity where emergency blood is unavailable are probably warranted.

A major limitation of this study was that these children were in another clinical trial that was investigating the safety and efficacy of volume expansion in children with severe malaria and features of impaired perfusion. It is thus not very clear whether fluid administration altered the outcome in terms of the number who would have required a blood transfusion. It is also unclear how the WHO transfusion guidelines would work where frequent re-assessment is not possible as this study found that 70% of children with severe anaemia who were stable at admission were eventually transfused.

## Conclusion

In conclusion, this study provides empirical data to support the rational use of blood transfusion in hospitalized children with severe malaria, which may help resolve the disparity between recommended policy and current clinical practise. When the guidelines are followed, there is neither an increase in mortality nor an increase in anaemia in children with severe malaria after discharge. Effective anti-malarial treatment at discharge prevents the development of rebound anaemia.

## Abbreviations

CI: confidence interval; HBV: Hepatitis B virus; HCV: Hepatitis C virus; HDU: High Dependency Unit; HIV: Human immunodeficiency virus; DSS: Demographic surveillance site; KDH: Kilifi district hospital; MUAC: mid upper arm circumference; SP: sulfadoxine-pyrimethamine; SSMA: severe symptomatic malaria anaemia; WHO: World Health Organization

## Authors' contributions

SOA enrolled patients to the studies, reviewed patients at follow-up, entered and analysed the data, and wrote the manuscript; OH helped analyse the data and reviewed the manuscript; AP enrolled patients to the studies, reviewed patients at follow-up, and reviewed the manuscript; RI enrolled patients to the studies, reviewed patients at follow-up, analysed the data and reviewed the manuscript; TW provided the DSS data for follow-up and reviewed the manuscript; CRJCN enrolled patients to the studies, analysed the data, and reviewed the manuscript; KM designed the study, analysed the data, reviewed all drafts of the manuscript. All authors read and approved the final manuscript.
